# Flexible side-biting aortic clamp via a centimetric proximal intercostal incision to facilitate proximal anastomosis during minimally invasive coronary artery bypass grafting

**DOI:** 10.1186/s13019-026-04108-7

**Published:** 2026-04-22

**Authors:** Taiqiang Chen, Xin Feng, Siqi Chen, Xiang Wei, Zemin Fang

**Affiliations:** 1 Division of Cardiothoracic and Vascular Surgery, Tongji Hospital, Tongji Medical College, Huazhong University of Science and Technology, 1095 Jiefang Ave, Wuhan, 430030 China; 2 Department of Cardiothoracic Surgery, The Affiliate Yili Friendship Hospital of Nanjing Medical University, Yining, China

**Keywords:** Minimally invasive cardiac surgery, Coronary artery bypass grafting, Proximal anastomosis

## Abstract

**Objective:**

Minimally invasive coronary artery bypass grafting (MICS CABG) via an anterolateral mini-thoracotomy represents a viable and safe approach. However, the proximal anastomosis of the saphenous vein to the ascending aorta can be technically challenging, particularly in cases with a prominent right ventricular outflow tract or pulmonary artery. In this report, we present an enhanced proximal anastomosis technique for minimally invasive coronary artery bypass grafting performed via intercostal thoracotomy.

**Methods:**

45 patients with a mean age of 64.6 ± 9.0 years underwent minimally invasive coronary artery bypass grafting with enhanced technique via intercostal thoracotomy between July 2021 and November 2023. Multilevel pericardial retractions were employed to laterally displace the heart and a flexible side-biting clamp was placed on the ascending aorta via a 1-cm incision in the left second intercostal space, enabling handsewn proximal anastomoses on the ascending aorta.

**Results:**

The mean number of grafts was 2.8 ± 0.5, with a total of 11 patients (24%) had 2 grafts, 32 patients (71%) had 3 grafts, and 2 patients (4%) had 4 grafts. Median operative time was 4.2 ± 0.7 h, mean intensive care unit stay was 1.5 ± 0.5 days, and mean in-hospital stay time was 9.1 ± 6.0 days. No in-hospital mortality occurred.

**Conclusion:**

This technique provides a reliable approach for overcoming technical limitations of proximal anastomosis in MICS CABG, especially when aortic access is problematic.

## Introduction

Coronary artery bypass grafting (CABG) is a widely favored treatment for severe coronary artery diseases. The conventional sternotomy remains considerable morbidity, including infection, post sternotomy pain, chronic sternal nonunion and slow return to full physical activity [1]. Minimally invasive coronary artery bypass grafting (MICS CABG) was developed to facilitate revascularization of all coronary artery territories through a small thoracotomy, demonstrating several advantages over sternotomy, such as reduced incision size, fewer postoperative complications, and expedited recovery [2, 3]. However, the adoption of MICS CABG has been limited in certain cardiac centers due to its inherent surgical complexities [4]. In various centers, a flexible side-biting clamp is employed for proximal anastomosis [4, 5]. Nonetheless, we observed that performing proximal anastomosis of great saphenous vein (GSV) grafts was challenging due to the restricted operational space and the considerable distance from the surgical incision to the stitching point during MICS CABG, particularly when the surgical view is obstructed by the protruding right ventricular outflow tract (RVOT) or pulmonary artery (PA). Over the years, some cardiac surgeons have advocated for alternative approaches, such as composite coronary artery bypass grafts and total arterial revascularization. Nevertheless, the conventional procedure of coronary bypass grafts, specifically the in situ left internal mammary artery (LIMA) and aorto-coronary saphenous vein grafts, continue to be the cornerstone of coronary bypass surgery in most centers [6]. Building on the technique of vein graft anastomosis, a novel method was developed for performing proximal anastomosis in selected cases of MICS CABG, which was found to be feasible and effective in clinical practice.

## Patients and methods

### Study population

This was a retrospective review of a consecutive series of 45 patients who underwent modified MICS CABG via left mini-thoracotomy by a single surgeon in our institution between July 2021 and November 2023. This study included patients with multivessel coronary artery disease (defined as ≥ 50% stenosis of the left main coronary artery or > 75% stenosis in other target vessels) who were referred for surgical revascularization. All patients underwent off-pump coronary artery bypass grafting (OPCABG) using MICS CABG, which is our preferred strategy for myocardial revascularization in eligible candidates. Additionally, patients were required to meet inclusion criteria: preoperative echocardiographic and computed tomography (CT) showed prominent right ventricular outflow tract (RVOT diameter > 40 mm on CT scan and or on echocardiography) or pulmonary artery (PA/AO > 1.1 on CT scan). Patients were excluded from the study if they required emergency surgery or had severe valve damage, ventricular aneurysms, congenital heart diseases, diffuse coronary artery disease, low left ventricular systolic function (ejection fraction ≤ 45%), left ventricular end-diastolic diameter ≥ 60 mm, presence of advanced atherosclerosis or calcification in aorta, thoracocyllosis on chest CT scans, severe pleural adhesions, or a history of severe pulmonary insufficiency.

The average age of patients was 64.6 ± 9.0. The mean body mass index (BMI) was 25.5 ± 3.7 kg/m². Of the 45 patients, 11 had heart failure and 5 had a history of stroke prior to admission. Fourteen patients had two-vessel disease and 31 had three-vessel disease. The mean left ventricular ejection fraction (EF) was 55.3 ± 11.2%. Fourteen patients were classified as having intermediate risk (EuroSCORE 3–6) and 5 patients for high operative risk (EuroSCORE ≥ 6). Table 1 presents the baseline and cardiovascular characteristics.

**Table 1:** Baseline parameters

Baseline parameters	*n* = 45
Age(years)	64.6 ± 9.0
BMI (kg/m^2^)	25.5 ± 3.7
Smoker	18(40%)
Hypertension	38(84%)
Diabetes mellitus	14(31%)
Hyperlipidemia	33(73%)
Previous Heart Failure	11(24%)
Previous Stroke	5(11%)
eGFR<60 ml/min	6(13%)
ESRD	2(4%)
NYHA	
Class I	2(5%)
Class II	25(55%)
Class III	18(40%)
Class IV	0(0%)
Cardiovascular parameters	
Symptoms:	
CCS1	2(4%)
CCS2	9(20%)
CCS3	27(60%)
CCS4	7(16%)
2-vessel disease	14(31%)
3-vessel disease	31(69%)
left main stenosis > 50%	16(36%)
Post-PCI	10(22%)
Left ventricular ejection fraction	55.3 ± 11.2
EuroSCORE	
Intermediate risk (EuroSCORE 3–6)	14(31%)
High risk (EuroSCORE ≥ 6)	5(11%)

Data are expressed as mean ± standard deviation or as absolute values with percentages

BMI (body mass index), eGFR (estimated Glomerular Filtration Rate), ESRD (end-stage renal disease), NYHA (New York Heart Association), CCS (Canadian Cardiovascular Society), Post-PCI (Post-percutaneous coronary intervention), EF (ejection fraction), EuroSCORE (European System for Cardiac Operative Risk Evaluation)

### Ethical standards

The study has been approved by the local ethics committee and has therefore been performed in accordance with the ethical standards laid down in the 1964 Declaration of Helsinki and its later amendments. The study was registered in the Institutional Review Board of our hospital (NO. TJ-IRB202411032) and written informed consent was obtained from all participants.

### Anesthesia

Intravenous administration is a standard technique in cardiac anesthesia. Anesthesia was induced and maintained using sufentanil (0.5 µg/kg/h), etomidate (0.25 mg/kg), pancuronium (0.1 mg/kg), sevoflurane, and propofol (3 mg/kg/h), with invasive monitoring conducted via standard arterial and venous lines. Esmolol was titrated intravenously to maintain a target heart rate of 60–80 beats per minute. A double-lumen endotracheal tube with an endobronchial blocker was employed to initiate single lung ventilation in all cases. Transesophageal echocardiography was not routinely employed during surgery, as the procedure was performed off-pump and relied primarily on surgical visualization and hemodynamic parameters.

### Surgical techniques

**Fig. 1:**
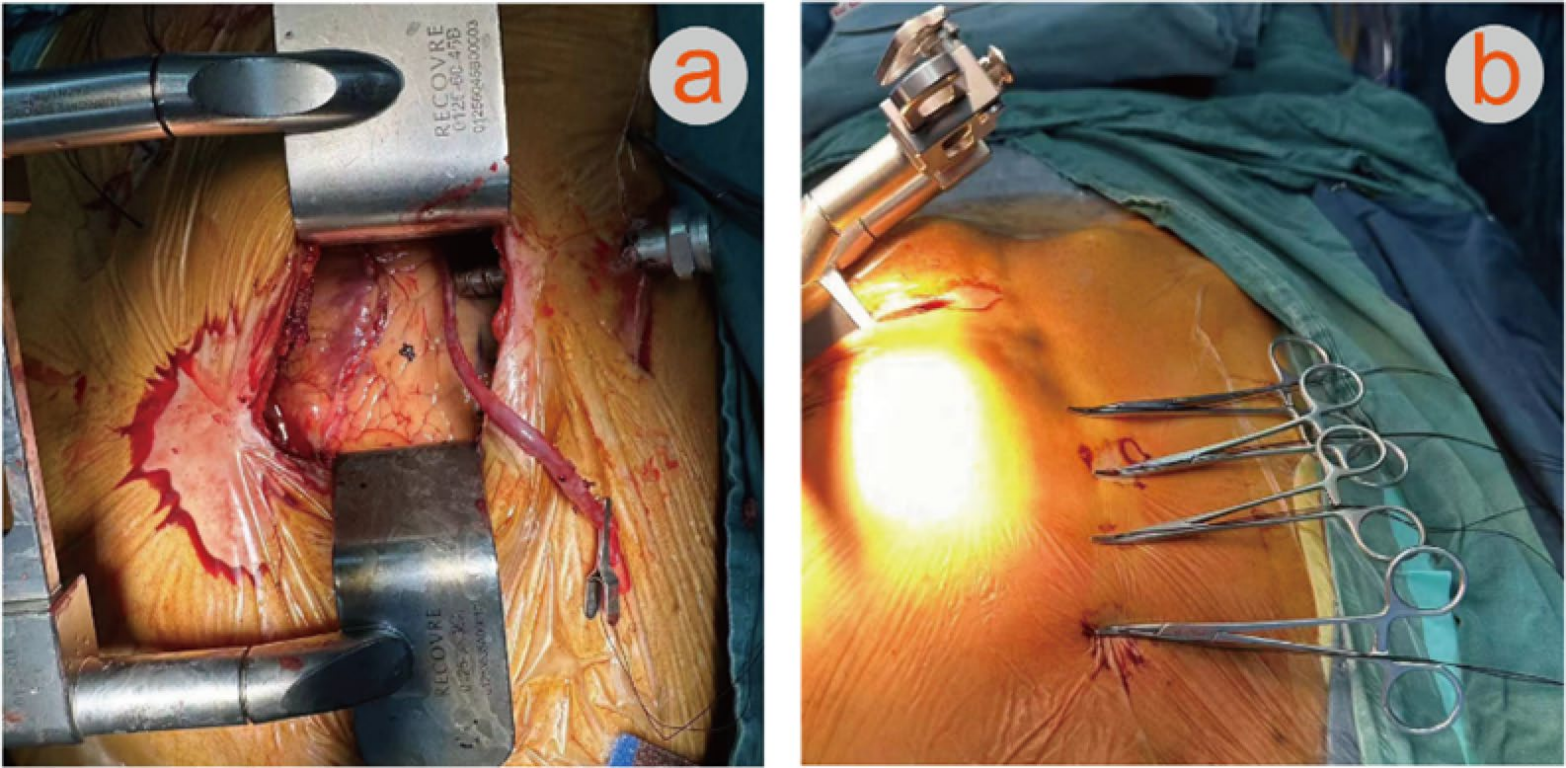
The site of main incision and skin puncture points for right pericardium suspension. **(a)** A 5–8 cm skin incision in the left fifth intercostal thoracotomy following ThoraTrak retractor position. **(b)** right pericardium suspension using stitches through the second to sixth intercostal parasternal spaces

**Fig. 2:**
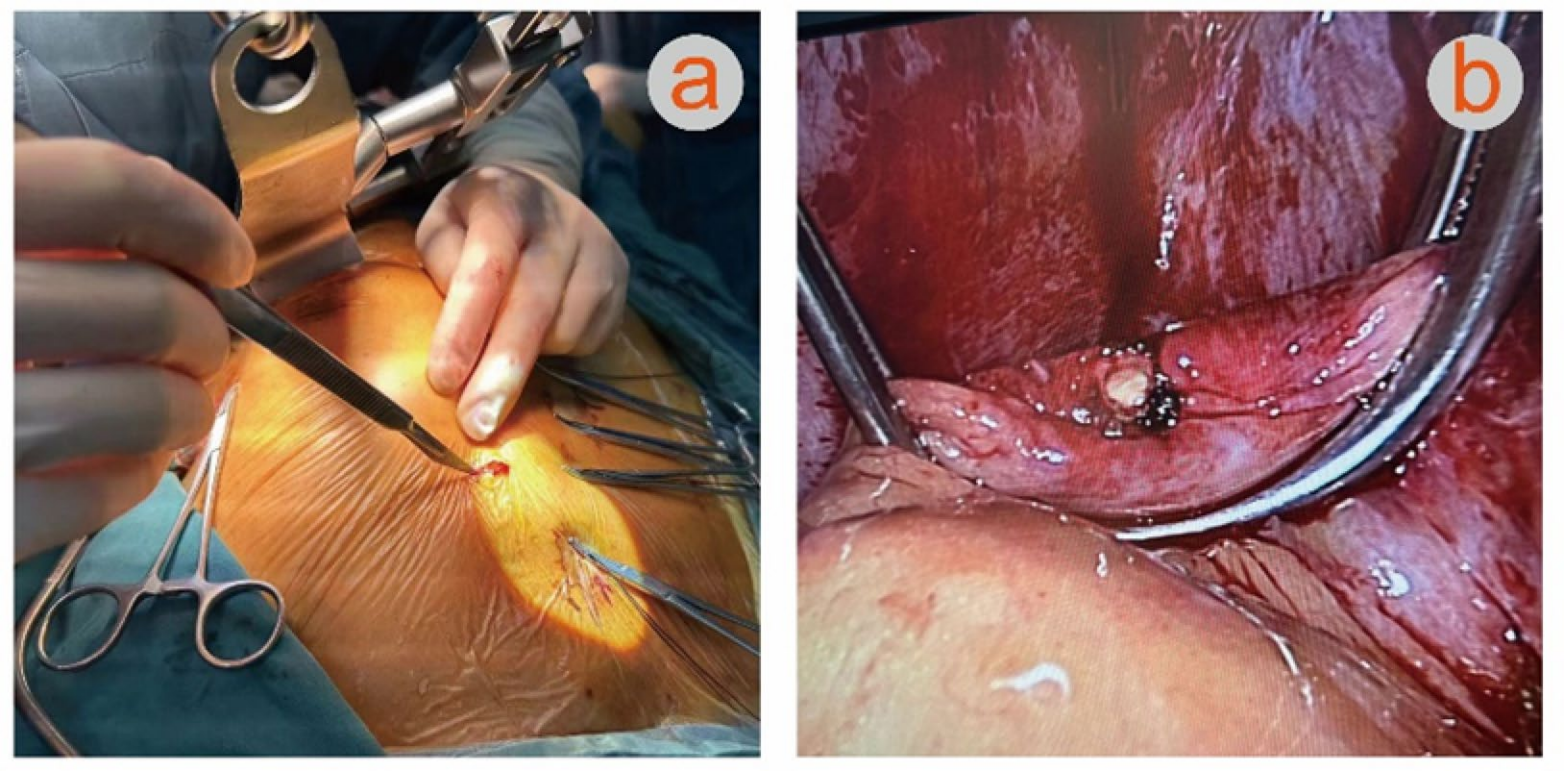
The site of extra incision and the position of flexible side-biting clamp. **(a)** 1-cm incision was made in the left second intercostal space. **(b)** flexible side-biting clamp was posited on the ascending aorta through minimal incision

**Fig. 3:**
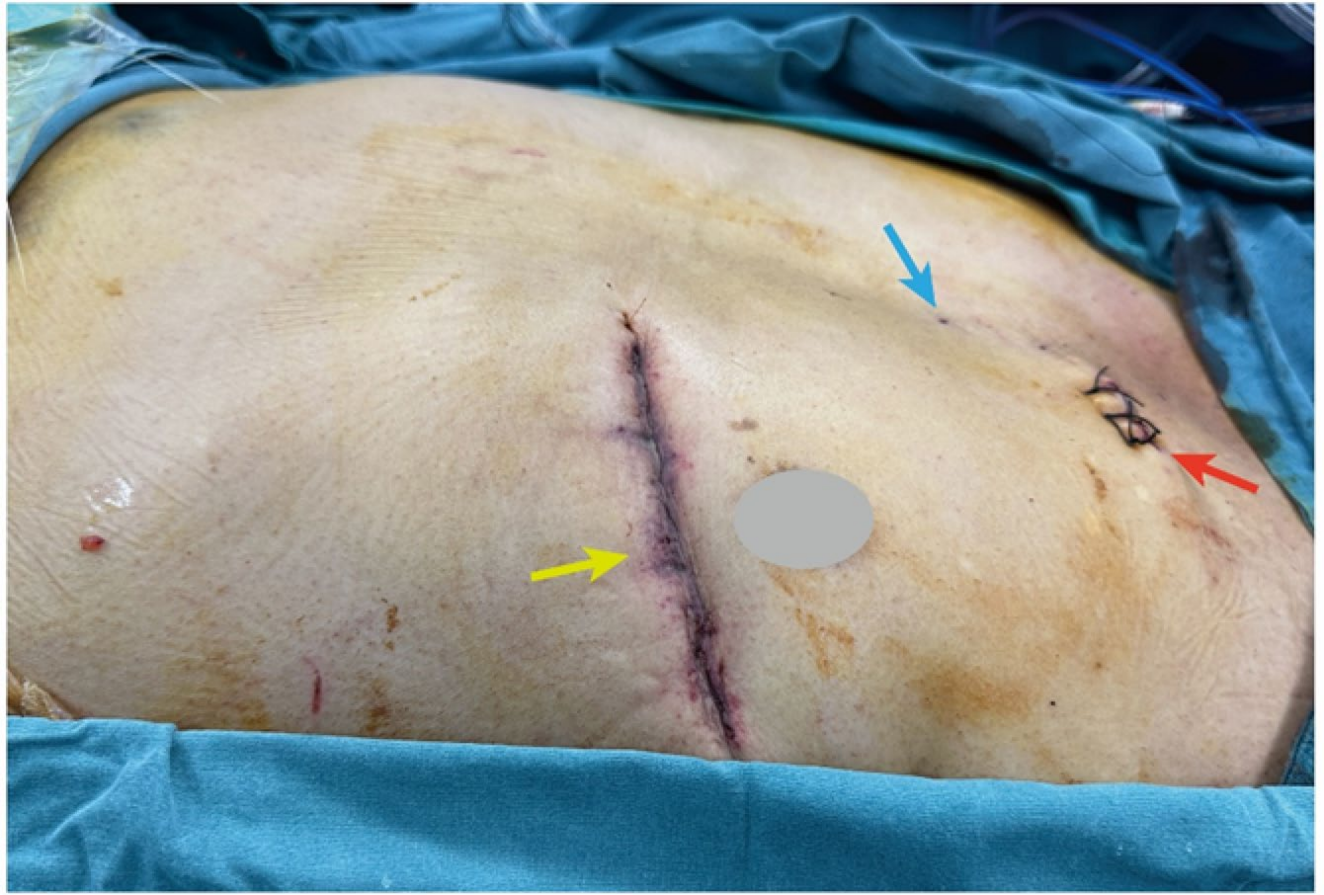
Overview of incisions. Main incision of left fifth intercostal thoracotomy (yellow arrow) and left second intercostal space for side-biting clamp insertion (red arrow) and skin puncture points of intercostal parasternal spaces (blue arrow)

Each patient was rotated 30° to the right by padding the left scapula. Left fifth intercostal thoracotomy about 6- to 8-cm was used to access myocardial territories. The ThoraTrak retractor (Medtronic, Minneapolis, MN, USA) was employed to open the thoracotomy (Figure 1a), with orientation directed cephalad and rightward towards the ascending aorta. GSV was harvested carefully from ankle to popliteal space rountinely and the left/right internal mammary artery (LIMA/RIMA) was harvested directly from the 1 st to the 6th intercostal space following systemic heparinization (1 mg/kg). The pericardium was incised from the ascending aorta to the left ventricular apex and the inferior vena cava. Afterwards three to four stitches through the second to sixth intercostal parasternal spaces were applied to retract and adequately suspend the right pericardium for laterally displacing the heart (Figure 1b). Next, the interval between aorta and pulmonary artery was cleared to facilitate effective side biting. Flexible side-biting clamp was placed on the ascending aorta via an extra 1-cm incision in the left second intercostal space, facilitating handsewn proximal anastomoses on the ascending aorta (Fig 2a and b). The LIMA/RIMA-left anterior ascending artery (LAD) anastomosis were prioritized with tissue stabilizer (Medtronic Octopus Nuvo). To access the left circumflex artery (LCX) territory, the heart was displaced cranially and to the right using a combination of deep pericardial gauze packing and a tissue stabilizer (Medtronic Octopus Nuvo), often accompanied by steep Trendelenburg positioning to maintain hemodynamics. For the right coronary artery (RCA) and its branches, the heart was gently rotated superiorly and to the left, again facilitated by the stabilizer and the aid of pericardial slings we established before. An intracoronary shunt (HTKD Medical HK-FL) was routinely inserted in all target vessels during distal anastomosis to preserve distal perfusion and maintain a bloodless field. Intraoperative blood flow post-vascular anastomosis was evaluated using transit-time flow measurement (TTFM). Before closure, a drainage tube was inserted into the left thoracic cavity via the 6th intercostal space (Figure 3).

### Statistical analysis

Continuous variables were summarized as mean ± standard deviation and categorical variables are expressed as frequencies or percentages. The data analysis was conducted using SPSS Statistics version 20.0 (IBM, Chicago, IL).

## Results

A total 126 distal anastomoses in 45 patients were performed, with an average of 2.8 ± 0.5 anastomoses per patient. The mean duration of surgery was 4.2 ± 0.7 h. The left anterior descending artery (LAD) territory was graft in all patients, the left circumflex artery coronary artery (LCX) coronary artery territory in 39 patients, and the right coronary artery (RCA) territory in 28 patients. RIMA harvesting was performed in two cases, because of young age with an inadequate LIMA, to achieve total arterial revascularization. A complex coronary reconstruction, involving open local coronary endarterectomy and reconstruction, was performed on one patient due to diffuse coronary target sclerosis. The surgical details are summarized below in Table 2.

**Table 2:** Operative data

Conduits used	
LIMA	43(96%)
RIMA	2(7%)
GSV	45(100%)
Revascularization territory of	
LAD	45(100%)
LCX	39(87%)
RCA	28(62%)
Number of distal anastomoses	2.8 ± 0.5
2	11(24%)
3	32(71%)
4	2(4%)
Coronary thrombendarterectomy	1(2%)
Conversion to open sternotomy	0(0%)
Length of surgery (hours)	4.2 ± 0.7

Data are expressed as mean ± standard deviation or as absolute values with percentages

LIMA: left internal mammary artery, RIMA: right internal mammary artery, GSV: great saphenous vein, LAD: left anterior descending artery, LCX: left circumflex artery, RCA: right coronary artery

Regarding adverse events, there were no instances of hospital mortality, superficial wound infection or postoperative stroke. Forty-one patients were ≤ 2 days intensive care unit (ICU) stay, with mean ICU stay time of 1.5 ± 0.5 days. Thirty-five patients were ≤ 8 days in-hospital stay, with mean in-hospital stay time of 9.1 ± 6.0 days. Whilst 8 out of 45 patients suffered from short-time postoperative atrial fibrillation (AF), and 4 patients received dialysis due to end-stage renal disease (ESRD) and acute kidney injury (AKI). Three patients experienced low cardiac output, and required Intra-aortic balloon pump (IABP) support. The rest two patients were reoperated due to bleeding, two experienced postoperative delirium, which was managed with expectant treatment, two developed pneumonia but recovered well, and one patient developed postoperative myocardial infarction. Postoperative outcome is given in Table 3.

**Table 3:** Postoperative adverse events and outcome

Adverse events	
Low cardiac output	3(7%)
Myocardial infarction	1(2%)
Revision due to bleeding	2(4%)
Dialysis	4(9%)
Delirium	2(4%)
Pneumonia	2(4%)
New onset of AF	8(18%)
Superficial wound infection	0(0%)
Stroke	0(0%)
IABP	3(7%)
Outcome parameters	
Time on ICU (days)	1.5 ± 0.5
≤ 2 day	41(91%)
In-hospital stay (days)	9.1 ± 6.0
≤ 8 days	35(78%)
In-hospital mortality	0(0%)

Data are expressed as mean ± standard deviation or as absolute values with percentages

IABP: Intra-aortic balloon pump; ICU: intensive care unit; AF: atrial fibrillation

## Discussion

**Fig. 4:**
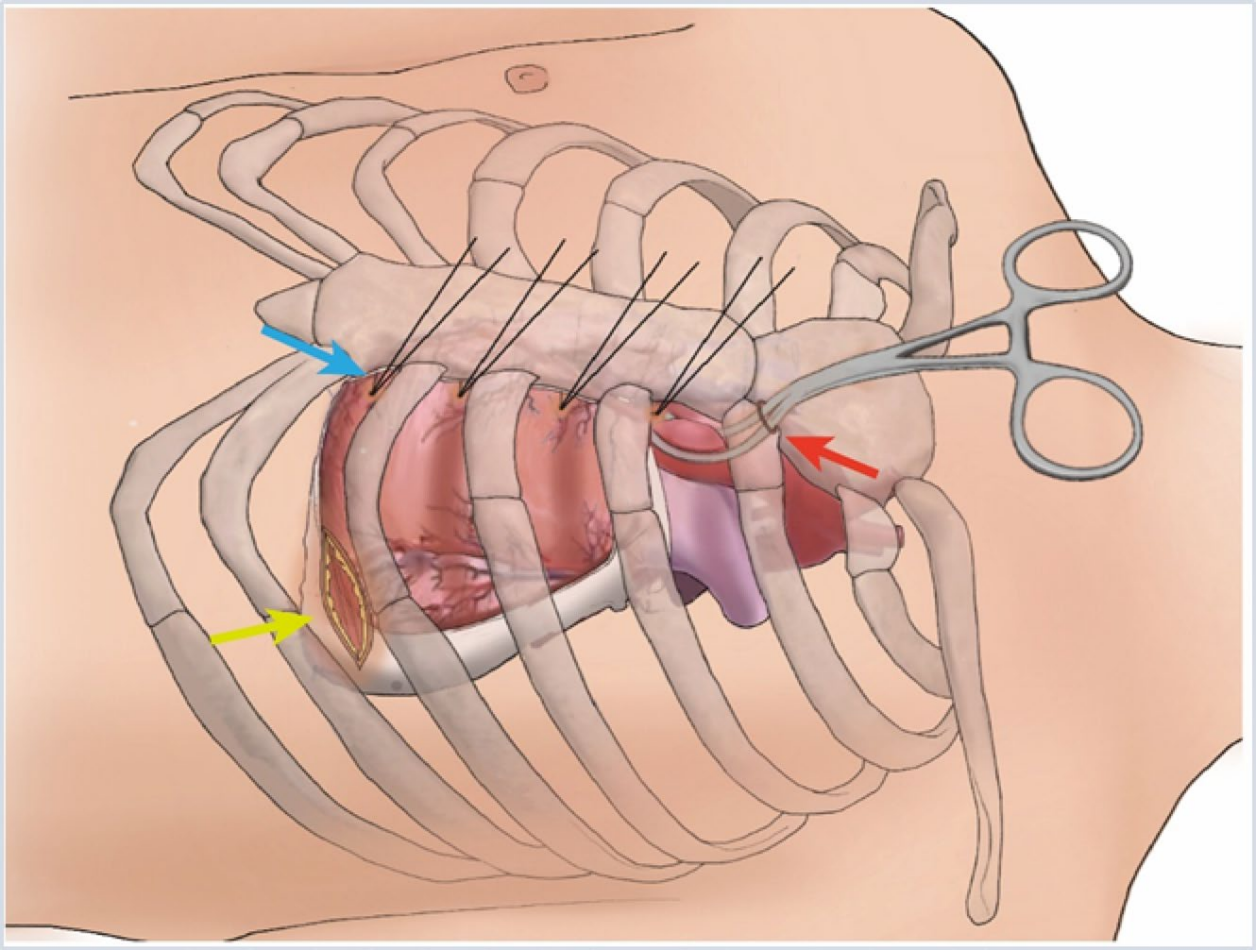
Schema of improved method. Side-biting clamp was inserted through 1-cm incision in the left second intercostal space (red arrow) and right pericardium was suspended using stitches through intercostal parasternal space (blue arrow) and main incision was located at the left fifth intercostal space (yellow arrow)

In this case series involving 45 enrolled patients, the modified minimally invasive CABG through a left anterior thoracotomy with a left second intercostal incision was technically feasible (Figure 4). Surgical outcomes were favorable with excellent proximal anastomoses and short operation time.

The first minimally invasive CABG procedure was conducted in 1994 by Benetti and Ballester [7]. It has been reported that minimal incision techniques offer several advantages over conventional sternotomy approaches, including enhanced cosmetic outcomes, a reduced risk of bleeding and wound infection, and expedited recovery, thereby making it a preferred option [2]. Consequently, the cost-effectiveness is reduced due to the shortened length of ICU stay and hospital stay with earlier discharged [8]. Stamou et al. demonstrated that MICS CABG also results in significantly fewer neuropsychological changes (0% vs. 19%, *p* = 0.03) and a lower incidence of postoperative atrial fibrillation (12% vs. 44%, *p* = 0.02) [9], indicating fewer postoperative complications and a faster recovery for patients. Following the implementation of MICS CABG, the duration of the surgery was reduced to 4.2 ± 0.7 h for surgeons. Concurrently, for patients, the average ICU stay was limited to 1.5 ± 0.5 days, and the mean in-hospital stay was reduced to 9.1 ± 6.0 days. It is noteworthy that three patients experienced hospital stays exceeding 15 days due to low cardiac output and the use of IABP. Our study confirmed that MICS CABG is both feasible and highly effective for patients requiring CABG.

Despite it’s potential, as previously mentioned, MICS CABG constitutes only a small fraction of the total CABG procedures and is performed at a limited number of centers. In both Europe and the USA, the adoption of minimally invasive CABG remains restricted to a minority of institutions [10]. It is estimated that approximately 5% of CABG procedures are conducted without sternotomy, with higher prevalence observed in Asia, Europe, and India [11]. Furthermore, minimally invasive CABG has received limited endorsement from international guidelines on myocardial revascularization [12, 13], likely due to the absence of large-scale randomized controlled trials, which has resulted in uncertainty regarding its benefits [10]. From a surgical perspective, the procedure remains technically demanding due to restricted heart exposure and limited operative space. To expand its applicability, a novel technique has been developed to address challenges associated with atypical heart positions, such as a prominent right ventricular outflow tract or pulmonary artery. Moreover, our successful experience indicates that revascularization using arterial and venous conduits is both feasible and scalable for MICS CABG, especially in moderate level centers.

While numerous studies have focused on surgical outcomes and angiographic graft patency in MICS CABG procedures, the technical challenges and their management have not been sufficiently addressed. For cardiac surgeons, vascular anastomosis demands greater surgical expertise, and performing off-pump MICS CABG for multi-vessel disease involves a significant learning curve. In fact, as plenty patients suffer from multiple coronary artery stenoses instead of single vessel disease, the advancement of multivessel CABG is imperative, so as MICS CABG. In 2009, McGinn et al. reported a dual-center study demonstrating that minimally invasive multivessel coronary revascularization is feasible in a large patient cohort, indicating broad applicability and excellent outcomes [14]. Moreover, recent reports from various institutions underscore the effectiveness of multivessel MICS CABG, demonstrating excellent clinical outcomes and mid-term graft patency comparable to the traditional sternotomy approach [2, 15, 16]. It reaches a consensus that internal mammary arteries are optimal for vascular bridging, and it’s proved that use of bilateral internal thoracic arteries in MICS CABG, employing Y and T grafts formed by radial artery or saphenous vein segments, has yielded favorable results [14, 17]. Composite coronary grafts, whether total arterial or arteriovenous, offer significant advantages by reducing manipulation of the ascending aorta. By avoiding the use of a partial occlusion clamp, this technique minimizes the risk of atheroembolism and subsequent stroke, which is a well-documented complication of aortic clamping in patients with atherosclerotic aortic disease [18]. Furthermore, mounting evidence suggests that constructing a Y-composite graft with the LIMA as the inflow not only reduces aortic trauma but also improves the long-term patency of saphenous vein grafts compared to traditional aorto-coronary anastomosis. The favorable hemodynamic profile and better size match between the LIMA and the vein graft are believed to contribute to this enhanced durability [19, 20]. However, this technique is not appropriate for patients with suboptimal internal mammary artery quality or in centers lacking advanced technical capabilities. Conversely, vein-to-aorta anastomosis plays a pivotal role in MICS CABG procedures, facilitating complete multivessel anatomical grafting akin to OPCABG. In this study, in addition to LIMA grafting, 45 cases of vein-to-aorta proximal anastomoses were performed, with the number of distal anastomoses being two or more, thereby demonstrating the feasibility of proximal anastomosis through a minimally invasive approach.

The side-biting clamp is an applicable device associated with proximal anastomosis in OPCABG. Consequently, there has developed various anastomosis-assist devices such as Heartstring, Enclose II, and PAS-Port showing benefit to patients with severe atherosclerotic plaque in the ascending aorta [21, 22]. These devices have been found to improve outcomes related to transient neurological complications [23], however, there is currently no relevant literature on their application in MICS CABG. Overall, the side-biting clamp remains a viable option in OPCABG. In MICS CABG, however, the use of a side clamp on the aorta presents challenges and risks due to limited surgical visibility and restricted aortic exposure. The proximal anastomosis space may be difficult to navigate if the view is obstructed by the right ventricular outflow tract or pulmonary artery, increasing the risk of improper clamping or potential injury to the right coronary ostium and aortic valve. To address this issue, several studies have suggested that displacing the right ventricular outflow tract left posteroinferiorly using an epicardial stabilizer can create additional space for proximal anastomosis. However, this technique may lead to transient pulmonary hypertension, which could potentially result in right heart injury [24–26]. Liu et al. proposed an alternative method involving the retraction of the pericardium near the ascending aorta, clearing the interval between the aorta and pulmonary artery, and placing a gauge on the right side of the aorta to improve exposure of the anastomosis site [27]. In this modified procedure, the right pericardium is effectively retracted and suspended with three to four stitches through the second to sixth intercostal parasternal spaces. Furthermore, a flexible side-biting clamp is introduced through a 1-cm incision in the left second intercostal space to achieve precise exposure of the ascending aorta, thereby facilitating hand-sewn proximal anastomoses (Figure. 4). Slightly lifting the clamp enhances accessibility to the anastomosis area. This modification simplifies the procedure, making it more manageable, especially for less experienced surgeons or in cases involving heart torsion.

Nevertheless, this study is subject to several limitations. Firstly, this is a retrospective, single-center, observational study and an initial experience report. A larger cohort study is warranted to further evaluate its potential. Secondly, the study is constrained by a small sample size and lacks mid- to long-term follow-up data. Therefore, the durability of the anastomosis created with this technique remains to be determined. We have now initiated a prospective registry with routine images at 1 and 3 years postoperatively for all future patients undergoing this technique. Thirdly, both no-touch technique and skeletonization technique were employed for GSV harvesting. Nevertheless, the adoption of a more minimally invasive approach, such as endoscopic harvesting, is a critical step for future refinement of this procedure. Fourthly, the additional surgical incision may elevate the risk of bleeding. Despite these limitations, the study introduces a valuable technique for proximal anastomoses MICS CABG. It potentially offers a practical solution for cardiac surgeons managing challenging cases involving difficult exposure of the ascending aorta.

## Conclusion

This initial experience suggests that minimally invasive coronary artery bypass surgery via minithoracotomy can be routinely reproduced safely. The method of using a flexible side-biting clamp on the ascending aorta via an incision in the left second intercostal space offers a secure solution to the technical challenges of proximal anastomosis in MICS CABG, especially in cases where the ascending aorta is hard-exposed.

## Data Availability

The data underlying this article will be shared on reasonable request to the corresponding author.
